# OP35 Multi-Criteria Decision Analysis Framework For Health Technology Prioritization In Mexico: Enhancing Decision-Making

**DOI:** 10.1017/S0266462325100937

**Published:** 2025-12-29

**Authors:** Verónica Gallegos-Rivero, Yesenia Ortíz-Montaño, Mariana Beatriz Pineda-López

## Abstract

**Introduction:**

Health technology assessment (HTA) is essential for informed, transparent, and efficient resource allocation in Mexico’s health system. Despite efforts by institutions like the General Health Council and the National Center for Health Technology Excellence (CENETEC), formal prioritization methods are lacking. This study aimed to develop a prioritization framework, based on the EVIDEM model, via a Delphi panel, integrating clinical, economic, ethical, and social criteria to enhance decision-making.

**Methods:**

Conducted between May and December 2024, this project was initiated by the Interinstitutional HTA Working Group to develop a preliminary multi-criteria decision analysis (MCDA) framework for prioritizing health technologies in Mexico. Phases included a systematic literature review, the formation of a multidisciplinary expert panel, and consensus rounds to select key criteria based on the EVIDEM model, ensuring non-redundancy and independence. The process and results were documented in a manuscript for publication in 2025, aiming to strengthen decision-making in Mexico’s health system.

**Results:**

In the first phase, 10 key studies were identified through a comprehensive search protocol, 60 percent of which utilized MCDA for prioritization. These studies averaged 19 relevant criteria, many of which were aligned with the EVIDEM framework, negating the need for new criteria. In the second phase, a multidisciplinary panel of 20 experts from key Mexican public health institutions was formed. Through two consensus rounds in the third phase, 10 of 25 fundamental criteria were selected, including disease severity, comparative effectiveness, costs, and evidence quality. Notably, 80 percent of the selected criteria aligned with the core EVIDEM model.

**Conclusions:**

The proposed MCDA framework marks an initial step toward a structured tool for prioritizing health technologies in Mexico. While validation is needed to confirm its applicability, implementation of the framework has the potential to optimize equitable, criteria-based decision-making, benefiting both the health system and the population.

